# Impact of Stunting on Development of Children between 1–3 Years of Age

**DOI:** 10.4314/ejhs.v32i3.13

**Published:** 2022-05

**Authors:** Muhammad R D Mustakim, Irwanto, Roedi Irawan, Mira Irmawati, Bagus Setyoboedi

**Affiliations:** 1 Department of Child Health, Faculty of Medicine Universitas Airlangga, Surabaya, Indonesia

**Keywords:** Children, Development, Stunting

## Abstract

**Background:**

Stunting occurs due to chronic malnutrition and is a major problem for children in developing countries. It is important to evaluate the impact of stunting on the development of children. This study aimed to investigate the impact of stunting on the development of children between 1–3 years of age.

**Methods:**

This cross-sectional study was conducted from July 2020 to March 2021 in Surabaya, Indonesia. A questionnaire and growth assessment were done, following the development measurement to stunted and non-stunted children who met the inclusion and exclusion criteria. Development was measured by the Denver Developmental Screening Test II (DDST-II), and Cognitive Adaptive Test/Clinical Linguistic & Auditory Milestone (CAT/CLAMS) scales.

**Results:**

Three hundred children are included in this study, consisting of 150 stunted and 150 non-stunted children. Stunted children had a higher risk to be suspected of delayed development compared to non-stunted children. The Crude Odd Ratio was 2.98, 4.24, 4.75 with the p-value 0.006, 0.001. and 0.001 respectively. The Adjusted Odd Ratio was 0.34, 0.24, 0.21 with p-value of 0.008, 0.001, and 0.001 respectively.

**Conclusion:**

Stunting is associated with suspected development delay among children 1–3 years of age. Initiatives related to prevention need to be established and nutrition advice needs to be provided.

## Introduction

Optimal nutritional care is one of the important factors needed to produce optimal growth and development, especially in children under five years of age. Nutritional deficiencies at that time will have an impact on growth and determine the further development of children (1). One form of malnutrition that is currently a global problem is stunting. Stunting is an impact that occurs due to chronic malnutrition and is a major problem for children in rural areas who experience developmental disabilities. The United Nations Children's Fund (UNICEF) states that the prevalence of stunting in the under-five population globally reaches 21.9% (2,3). Based on these data, the highest number of stunting cases is in Africa and Asia, including Indonesia. Indonesia's basic Health Research survey shows that more than a third of children (30.8%) of the population under five are stunted. This condition must be overcome.

The most frequent form of child malnutrition is stunting (4). Malnutrition in early life might cause inflammation, changes in leptin levels, and increased glucocorticoids resulting in epigenetic changes. These changes may cause neurodevelopmental disorders, changes in neurogenesis and cell apoptosis as well as dysfunction of synapses resulting in developmental delay (5). It was concluded that malnutrition affects areas of the brain involved in cognition, memory and locomotor skills (6–8). The relationship between stunting and cognitive function also has been demonstrated (9). Children who were persistently stunting had significant lower cognition than children who were not stunted (10). The effects of stunting on neurocognitive function are severe. Stunted children have stunted brains and live stunted lives, preventing entire communities from developing (4). Growth stalling often starts in utero and lasts for at least the first two years after birth. Human development is hampered by the severe irreparable physical and neurocognitive harm that comes with stunted growth. Children with stunting have a 3.6 times higher risk of cognitive impairment than children without stunting (11). One effort that can be done to prevent stunting is to improve the nutritional intake for pregnant women and toddlers. This practical approach can be applied to prevent stunting, with regards to reducing the prevalence of child developmental disorders. In connection with the negative impact of stunting on children's development, it is necessary to evaluate the development of children who are stunted. Indonesia has the fifth-highest level of stunting in children among countries (3). This condition was exacerbated by the Covid-19 pandemic which had an impact on the economy of the community, causing an increase in the number of cases of stunting due to a decrease in the ability of parents to meet children's nutritional needs. The high incidence of stunting surely carries high burden for the country. Research conducted to assess the effect of stunting on children's development has not been done much. This condition triggers the researchers to further observe the impact of stunting on the development of children, especially in children between 1–3 years of age.

## Methods

We conducted a cross-sectional study from July 2020 to March 2021. The population in this study were children aged 1 to 3 years old who were in Surabaya, Indonesia. We used consecutive sampling by collecting the participants with stratified random sampling. The participants of this study were stunted and non-stunted children who met the inclusion and exclusion criteria. We collected the participants from Primary Health Care which are located in Surabaya, Indonesia. We categorized the participants of the study into two groups, stunted and non-stunted children. The inclusion criteria of stunted children are 1) children aged from 1 to 3 years old who diagnosed stunted in Primary Health Care 2) proportionate short stature 3) has a history of chronic malnutrition, and 4) parents are willing to sign an informed consent to participate in the study. The definition of stunted children was based on WHO criteria with a length/height below - 2 Standard Deviation who have experienced linear growth faltering (12). The inclusion criteria of non-stunted children are as follows: 1) healthy children aged from 1 to 3 years, 2) parents are willing to sign an informed consent to participate in the study. The exclusion criteria of the study are: 1) children have a history of congenital anomaly, 2) has a history of other chronic diseases 3) has severe neurological deficits, and 4) children with short stature due to genetic or hormonal disorders. The ethical clearance of this study was approved and issued by the Health Research Ethics Committee, Faculty of Medicine, Universitas Airlangga.

Data collection was carried out by visiting and measuring the patients one by one after obtaining permission from the Surabaya Public Health Department. The researcher has been swabbed PCR-COVID19 and declared negative. The subject and their family living in the same house have been swabbed Antigen-COVID19 and declared non-reactive. After the parents are willing to sign the informed consent. We deliver a brief explanation regarding the study and we gave a questionnaire for parents to be filled in. The questionnaire contains the data on the characteristics of participants including chronological age, gender, history of breastfeeding, socio-economic, and parental education. A questionnaire was also conducted to ensure that no exclusion criteria were found in the study participants. Following the questionnaire, we started to measure the growth in children. After the growth measurement was done, the children will be assessed for development using 2 measuring instruments. The first measuring instrument was the Denver Developmental Screening Test (DDST-II) to measure fine motor, gross motor, and personal-social development (13). The participants are categorized as suspects if they have one or more delays and/or two or more cautions, while considered as normal if they have no delay in any domain and no more than one caution. The second instrument was the Cognitive Adaptive Test/Clinical Linguistic & Auditory Milestone (CAT/CLAMS) scales to measure cognitive function (14). We categorized the participants as suspect if they have scored below 85, whereas the normal participant was classified if they have scored above or equal to 85.

Descriptive analysis was performed using statistical measures of mean ± standard deviation (SD), number (percentage), median, and interquartile range (IQR). Mann-Whitney U tests are used for the data that are not normally distributed. The bivariate analysis was tested to seek the correlation between stunting and the children's development. The Crude Odd Ratio (COR) was obtained using the Chi-Square test and Adjusted Odd Ratio (AOR) was acquired using Binary Logistic Regression to perceive the determinant factors of stunting in children's development. The analysis of the data was done with IBM SPPS Statistic Version 23. A p-value < 0.05 was considered statistically significant.

## Results

A total of 300 participants were included in this study. We divided the participants into two groups, 150 stunted children and 150 non-stunted children. The mean age of the children is 23.12 months ranging from 12 to 35 months. The data of socio-demographic and characteristics of all participants are presented in Table 1. The mean difference of actual height in stunted children and non-stunted children is 6.78 cm. There are significant differences between stunted and non-stunted children especially from the history of exclusive breastfeeding and physical appearance such as body weight, height, and head length (p = 0.001) (Table 1). In Table 1, we also compared the risk factors that might be the causes as to why children are stunted. The children who are categorized as the suspect in delayed development are found mostly in stunted children. Significant differences, mostly on motor function (both in fine and gross motor function), are found in development of stunted and not stunted children (Table 2).

**Table 1:** Socio-demographic and characteristics of stunted and non-stunted children

	Non-stunted	Stunted	*p*
	N = 150	N = 150	
**Characteristic of children**			
Child's age (months)	22.91 ± 6.26	23.33 ± 6.54	0.502^a^
Sex			0.562^b^
Female	64 (42.7)	70 (46.7)	
Male	86 (57.3)	80 (53.3)	
Gestational age (weeks)	38 [36 – 29]	38 [25 – 42]	0.052^b^
Term	147 (98)	139 (92.7)	
Preterm	3 (2)	11 (7.3)	
Type of delivery			
Spontaneous	120 (80)	88 (58.7)	
Caesarian section	30 (20)	62 (41.3)	
Birth weight (gram)	3030 ± 230.11	2953.27 ± 463.62	0.001^b^*
≥ 2500 gram	149 (99.3)	134 (89.3)	
< 2500 gram	1 (0.7)	11 (10.7)	
Mother's age when pregnant	26.53 ± 6.60	26.94 ± 5.38	0.913^a^
Exclusive breastfeeding			0.001^b^*
Yes	139 (92.7)	102 (68)	
No	11 (46.3)	48 (32)	
Actual weight (kg)^a^	10.59 ± 1.96	9.67 ± 1.86	0.001^a^*
Actual height (cm)^a^	85.49 ± 8.07	78.71 ± 6.02	0.001^a^*
Actual head length (cm)^a^	47.76 ± 2.78	46.88 ± 3.04	0.001^a^*
Socio-economic of family			
Father's education			0.100^b^
Primary School	0 (0)	6 (2)	
Middle School	18 (12)	16 (10.7)	
Secondary School	66 (44)	66 (44)	
Tertiary education	66 (44)	62 (41.3)	
Mother's education			0.183^b^
Primary School	2 (1.3)	8 (5.3)	
Middle School	16 (10.7)	16 (11.3)	
Secondary School	94 (62.7)	82 (54.7)	
Tertiary education	38 (25.3)	44 (29.3)	
Family income			0.491^b^
< regional minimum wage	113 (75.3)	119 (79.3)	
≥ regional minimum wage	37 (24.7)	31 (20.7)	
Parenting			0.376^b^
Parents	135 (90)	129 (86)	
Other family member	13 (8.7)	20 (13.3)	
Babysitter	2 (1.3)	1 (0.7)	

Data are presented as N (%), Mean ± Standard Deviation, Median [Interquartile range]; The data are not normally distributed

a Mann-Whitney U test

b Chi-Square are used

* a p-value < 0.05 was statistically significant.

**Table 2:** The results of development on stunted and non-stunted children between 1–3 years of age

	Non-stunted	Stunted	*p*
	N = 150	N = 150	
**DENVER II Developmental Status**			
Normal	141 (94)	126 (84)	0.006*
Suspect	9 (6)	24 (16)	
**CAT/CLAMS**			
CAT			0.001*
Normal	122 (81.3)	76 (50.7)	
Suspect	28 (18.7)	74 (49.3)	
CLAMS			0.001*
Normal	114 (76)	60 (40)	
Suspect	36 (24)	90 (60)	

* a p-value < 0.05 was statistically significant; Chi-square analysis was used for the analysis.

Table 3 summarizes the COR and AOR of developmental function. The table shows that stunted children had a higher risk for children to experience the development delay compared to non-stunted children COR: 2.984, p = 0.006; AOR: 0.335, p = 0.008. We also found the same situation in the CAT/CLAMS instrument measuring the development of stunted children with p = 0.001 (Table 3). Stunted children can have the risk of experiencing delayed non-language visual-motor skills (CAT) by 4.242 times compared to non-stunted children (AOR 0.236; p = 0.001). This case was also the same with language skill (CLAMS), stunting for children have the risk of at least 4.750 times experiencing delayed language skills compared to not stunted children (AOR 0.211; p = 0.001). Table 4 also present determinant factors which contributed in suspect delayed development.

**Table 3:** Bivariate and multivariate results between stunted and delayed development

	Crude OR	95% CI (Lower – Upper)	*p*	Adjusted OR	95% CI (Lower – Upper)	*p*
**DENVER II**						
Stunted	2.984	1.337 – 6.660	0.006*	0.335	0.150 – 0.748	0.008*
Yes						
No						
**CAT**						
Stunted	4.242	2.520 – 7.141	0.001*	0.236	0.140 – 0.397	0.001*
Yes						
No						
**CLAMS**						
Stunted	4.750	2.889 – 7.809	0.001*	0.211	0.128 – 0.346	0.001*
Yes						
No						

Crude OR used Chi-Square Test; Adjusted OR used Binary Logistic Regression;

* a p-value < 0.05 was statistically significant.

**Table 4:** The Clinical risk factors of children between 1–3 years old compared with development Adjusted OR used Binary Logistic Regression

Variable	Adjusted OR	95% CI (Lower – Upper)	*p*
**DENVER II**			
Birth weight (gram)	0.793	0.202 – 3.118	0.740
Gestational age (weeks)	1.145	0.229 – 5.720	0.869
Father's education	0.820	0.318 – 2.113	0.681
Mother's education	0.465	0.200 – 1.082	0.075
**CAT**			
Birth weight (gram)	0.592	0.202 – 1.743	0.342
Gestational age (weeks)	0.715	0.227 – 2.246	0.565
Father's education	1.491	0.800 – 2.779	0.209
Mother's education	0.431	0.227 – 0.816	0.010*
**CLAMS**			
Birth weight (gram)	0.510	0.175 – 1.482	0.216
Gestational age (weeks)	0.858	0.272 – 2.709	0.794
Father's education	0.990	0.548 – 1.788	0.973
Mother's education	0.497	0.270 – 0.915	0.025*

* a p-value < 0.05 was statistically significant.

## Discussion

This study found a significant relationship between stunting with development of children aged 1–3 years. Delayed development in stunted children is caused by nutritional deficiencies that occur early in life. The intrauterine period is the first stage of the critical period of child development. In that period, nutritional factors play an important role in the maturation process of the central nervous system (15). A study conducted on animals and humans showed that the chronicity, time, and severity of nutritional deficiencies have an effect on brain development that will have an impact on subsequent developmental abilities (16). We found that children who were stunted had a greater risk of experiencing delays in motor development, both gross and fine motor. If the muscle mechanism has not developed properly, motor movement will not be perfect. This eventuality occurs in children with stunted development abnormalities, striped muscle or striated muscle that regulates subconscious actions that develop at a slower rate, and there can be no coordinated voluntary action before the child is in normal condition (17).

These results are coherent with a study that shows that acute malnutrition that occurs in children aged 1 year is associated with developmental delays in gross motor, fine motor, social, and language interactions (18). Similar results were also obtained from a study on stunted children aged 9–24 months, which showed that the fine motor skills of stunted children were worse than not stunted children (19). Another study in Bangladesh showed that stunted children had lower motor scores than not stunted children. They also reported that stunting at <2 years of age was a predictor of delays in motor development (20).

As previously described, several studies stated that the delays in motor development experienced by stunted children were mainly related to the maturation process of the central nervous system. However, low height in stunted children was also a factor in the delay (15). A study in 2013 showed that there were significant associations between gross motor development and anthropometry measures at 12 months. Increment of one height z-score in terms of the decrease in the average time taken to ‘lie to sit’ of 1.80 months (p = 0.03) (21). A meta-analysis study conducted in 2015 shows a significant correlation between height and motor skills in children in low-middle income Countries with a correlation value of 0.24. The meta-analysis also shows that height measures significantly affect children's walking ability earlier (p <0.05) (22).

We also found that stunted children also had significantly lower cognitive scores than those who were not stunted. The results of the present study are similar to a Peruvian study that showed that when compared to their non-stunted peers, children who were stunted from the age of 6 months to 6 years or stunted during childhood had considerably worse scores on cognitive capabilities (verbal vocabulary and quantitative test scores) at the age of 4.5 to 6 years (22). A study in Ethiopia found that stunting harms the cognitive development of children at the age of five and eight years old (11). The quantitative assessment for cognitive function score was higher for non-stunted children with 8.60 percentage points of difference (in favour of non-stunted children) with a significant statistical value (p <0.01) (11). The link between linear growth and cognitive development in the first two years of life was discovered in a metaanalysis of 29 LMICs, but the meta-analysis was unable to integrate educational, follow-up, and environmental data to describe the link between stunting and cognitive impairment in children (22). However, whether these changes are permanent or reversible is still a point of contention in the literature. A recent study showed that children who experienced catch-up growth had no difference in verbal vocabulary and quantitative test scores compared to nonstunted children. The authors concluded that children can recover from early nutritional deficiencies and improve their cognitive performance (23).

Our analysis showed that gestational week was positively associated with gross motor skills. This result is in line with a study in 2014 that stated that small for gestational age infants are more susceptible to motor developmental delay but may decrease with increasing age when compared to term infants (24). This is in contrast with the study done by Vungarala and Rajeswari (2018) that found no significant correlation between gestational age and motor development at eight months of corrected age (25). This study confirms that other factors such as the father's education are also important determinants of cognitive function. A father's education was positively associated with a child's cognitive function (26). This phenomenon is associated with better verbal activity skills in fathers who have higher education (i.e., college-level) (27). A systematic review regarding the impact of father involvement on children's cognitive skills showed that children whose fathers are content with their parenting and financially supportive of their families have superior linguistic and cognitive skills, according to research. In this study, the father's education is one of the determinants that affect children's cognitive function. Another study in Ecuador stated that parents' education has important implications for child cognitive development. The schooling and vocabulary levels of mothers were strong predictors of the cognitive development of young children (28).

In this study, we also found that fewer children in the stunting group received exclusive breastfeeding than the not stunted group. Breastfeeding supplies the newborn with colostrum, which is high in nutrients, as well as antibodies, which are essential for the formation of the gut microbiota and immune system. Any additional drink or food introduced before the age of four months is linked to an increased risk of gastrointestinal disorders, which can lead to growth retardation, nutritional deficiencies, and predisposition to infectious diseases in the first two years of life. The link between exclusive breastfeeding and length-for-age and weight-forage was verified in a study by Kuchenbecker et al. (p= 0.001) (29). Exclusively breastfed infants under the age of six months were taller, heavier, and less likely to be stunted than non-exclusively breastfed infants.

We also found that stunted children also had lower birth weight than those who were not stunted. History of birth weight less than 2500 grams were found to be more in the stunting group than the control group. These results are also consistent with a study, which stated that birth weight less than 2500 grams was a risk factor for stunting in children aged 6–24 months (AOR = 5.3) (30). Similar studies were also found in studies conducted in Ethiopia which stated that birth weight is a strong predictor in determining the anthropometric status of children in the future, this is because most babies with low birth weight are not able to catch up with normal growth in childhood (11).

This study is the first that analyzes the Impact of Stunting on the Development of Children between 1–3 Years of Age while in the COVID-19 pandemic. However, we admit that this study has limitations concerning other factors that are not properly discussed. Certain characteristics, which may be major predictors of poor motor and cognitive skills in children throughout their early years of life, were not evaluated. For example, maternal health, growth hormone, nutritional deficiency, lack of kid stimulation, violent exposure, and some environmental factors.

Stunting is associated with delayed development among children 1–3 years of age, mostly in motor function. Initiatives related to prevention need to be established and nutrition advice needs to be provided. Our study also confirms that other factors such as the gestational week and the father's education are also important determinants of development. Periodic screening is necessary, especially in children aged 1–3 years. Stunting prevention programs must be one of the top priorities for health workers in services close to the community, especially primary health care. If the child shows symptoms of stunting, they are immediately referred to a paediatrician to be able to improve their nutritional status.
